# Dental biomaterials redefined: molecular docking and dynamics-driven dental resin composite optimization

**DOI:** 10.1186/s12903-024-04343-1

**Published:** 2024-05-13

**Authors:** Ravinder S. Saini, Rayan Ibrahim H. Binduhayyim, Vishwanath Gurumurthy, Abdulkhaliq Ali F. Alshadidi, Lujain Ibrahim N. Aldosari, Abdulmajeed Okshah, Mohamed Saheer Kuruniyan, Doni Dermawan, Anna Avetisyan, Seyed Ali Mosaddad, Artak Heboyan

**Affiliations:** 1 Department of Dental Technology, COAMS, King Khalid University, Abha, Saudi Arabia; 2 Department of Prosthodontics, College of Dentistry, King Khalid University, Abha, Saudi Arabia; 3 Applied Biotechnology, Faculty of Chemistry, Warsaw University of Technology, Warsaw, Poland; 4 Department of Therapeutic Stomatology, Faculty of Stomatology, Yerevan State Medical University after Mkhitar Heratsi, Yerevan, Armenia; 5 Department of Research Analytics, Saveetha Dental College and Hospitals, Saveetha Institute of Medical and Technical Sciences, Saveetha University, Chennai, India; 6 Student Research Committee, School of Dentistry, Shiraz University of Medical Sciences, Qasr-E-Dasht Street, Shiraz, Iran; 7 Department of Prosthodontics, Faculty of Stomatology, Yerevan State Medical University after Mkhitar Heratsi, Str. Koryun 2, 0025 Yerevan, Armenia; 8 Department of Prosthodontics, School of Dentistry, Tehran University of Medical Sciences, North Karegar St, Tehran, Iran

**Keywords:** Biomaterial, Dental resin composite, In silico, Molecular docking, Molecular dynamics

## Abstract

**Background:**

Dental resin-based composites are widely recognized for their aesthetic appeal and adhesive properties, which make them integral to modern restorative dentistry. Despite their advantages, adhesion and biomechanical performance challenges persist, necessitating innovative strategies for improvement. This study addressed the challenges associated with adhesion and biomechanical properties in dental resin-based composites by employing molecular docking and dynamics simulation.

**Methods:**

Molecular docking assesses the binding energies and provides valuable insights into the interactions between monomers, fillers, and coupling agents. This investigation prioritizes SiO_2_ and TRIS, considering their consistent influence. Molecular dynamics simulations, executed with the Forcite module and COMPASS II force field, extend the analysis to the mechanical properties of dental composite complexes. The simulations encompassed energy minimization, controlled NVT and NPT ensemble simulations, and equilibration stages. Notably, the molecular dynamics simulations spanned a duration of 50 ns.

**Results:**

SiO_2_ and TRIS consistently emerged as influential components, showcasing their versatility in promoting solid interactions. A correlation matrix underscores the significant roles of van der Waals and desolvation energies in determining the overall binding energy. Molecular dynamics simulations provide in-depth insights into the mechanical properties of dental composite complexes. HEMA-SiO_2_-TRIS excelled in stiffness, BisGMA-SiO_2_-TRIS prevailed in terms of flexural strength, and EBPADMA-SiO_2_-TRIS offered a balanced combination of mechanical properties.

**Conclusion:**

These findings provide valuable insights into optimizing dental composites tailored to diverse clinical requirements. While EBPADMA-SiO_2_-TRIS demonstrates distinct strengths, this study emphasizes the need for further research. Future investigations should validate the computational findings experimentally and assess the material's response to dynamic environmental factors.

## Introduction

Dental resin-based composites have been widely adopted in modern restorative dentistry, primarily due to their superior aesthetic characteristics and capacity to establish direct adhesive bonds with tooth structures [1]. These materials, consisting of a matrix comprising polymerizable monomers and inorganic fillers, offer aesthetically pleasing and biocompatible options for tooth restoration [2]. The matrix generally constitutes a three-dimensional dental resin network incorporating bisphenol-a-glycidyl methacrylate (Bis-GMA) blended with triethylene glycol dimethacrylate (TEGDMA) monomers [3]. However, despite their advantages, dental resin composites have limitations. These limitations include challenges related to adhesion to tooth surfaces and suboptimal biomechanical properties compared to traditional restorative materials such as amalgam or ceramics [4, 5]. Adhesion is paramount for the long-term success of dental restoration [6]. Microleakage at the composite-tooth interface can result in secondary caries and restoration failure. Additionally, dental composites often exhibit inferior flexural strength and fracture toughness compared to alternatives, limiting their ability to withstand rigorous masticatory forces in the oral cavity [7, 8].

Optimizing adhesion to tooth structures is pivotal to preventing microleakage and secondary caries, common precursors of restoration failure and patient discomfort [9, 10]. Incorporating inorganic fillers, such as SiO_2_, CaCO_3_, CeO_2_, and HCa_5_O_13_P_3_, plays a crucial role in enhancing the properties of dental resins. One primary benefit is reducing polymerization shrinkage, a common challenge in dental resin materials. Introducing these fillers mitigates the overall volume contraction during polymerization, improves dimensional stability, and minimizes the risk of gaps or microleakage at the restoration-tooth interface [11–14]. Furthermore, enhancing biomechanical properties in dental resin composites, encompassing parameters such as Young’s modulus, shear modulus, and flexural strength, is paramount in ensuring their longevity and resilience in the demanding oral environment [7]. Young’s modulus, a measure of material stiffness, plays a pivotal role in determining how a dental composite responds to external forces. For dental restorations, a high Young’s modulus is of particular interest in scenarios where the restoration is directly subjected to chewing stress. This is crucial to prevent the risk of inducing cracks in the tooth structure, ensuring the restoration can withstand the demanding forces exerted during mastication [15–18]. On the other hand, the shear modulus represents a material’s resistance to deformation under shear stress. In dental applications, shear force is common during biting and chewing. A higher shear modulus implies greater resistance to shape distortion under shear stress, contributing to the overall structural integrity of dental composites [19–21]. Furthermore, flexural strength is a critical indicator of dental material performance, dictating its ability to endure bending forces encountered during mastication [22–24].

These challenges underscore the pressing need for innovative strategies to enhance the performance of dental resin composites. This research paper embarks on a pioneering journey to address these limitations by applying molecular dynamics-driven optimization. Molecular docking and molecular dynamics simulations have evolved into formidable tools in drug discovery and materials science [25, 26]. When applied to dental resin composites, these computational techniques present an opportunity to identify candidate modifiers, such as adhesion promoters or reinforcing agents, and predict their efficacy in ameliorating adhesion and biomechanical properties.

As a fundamental component of this research, molecular docking entails the computational anticipation of binding interactions among molecules. Dental resin composites allow the evaluation of composite constituents’ interaction with tooth structures or other pertinent biomolecules [27]. This knowledge, gleaned from molecular docking simulations, can be used as a compass for selecting modifiers capable of enhancing adhesion. In contrast, molecular dynamics (MD) simulations offer a dynamic perspective on molecular interactions over time [28]. By subjecting composite materials to dynamic conditions that mimic the oral environment, researchers can acquire insights into the evolving nature of intermolecular interactions. This dynamic understanding holds the potential to unveil avenues for optimization in the pursuit of improved adhesion and biomechanical properties.

MD simulations have emerged as a pivotal atomic-level method in dental materials, offering a bottom-up approach for characterizing and predicting material properties. This study employed MD to investigate the reinforcement effects and mechanisms within a dental resin composite model that integrates monomers, fillers, and coupling agents. The objective is to furnish a nanoscale understanding of the structures and performance of dental resin systems, specifically focusing on their impact on the biomechanical properties of dental resins. By scrutinizing the interactions at the atomic and molecular levels, this study seeks to unravel the intricate details governing the behavior of these composite materials. This approach promises to provide valuable insights into the nuanced world of dental materials, paving the way for informed advancements and innovations in restorative dentistry.

## Methods

The methodology employed in this research represents a comprehensive and systematic approach to investigating and optimizing the adhesion and biomechanical properties (Young’s modulus, shear modulus, and flexural strength) of dental resin-based composites using molecular docking and dynamics-driven simulations. This section provides a detailed overview of the critical steps and their importance in achieving the research objectives.

### Selection of dental resin-based composites for study

The initial step in this research involved careful selection of dental resin-based composites to form the basis of the study. These composites were selected because of their diverse formulations and properties available on the market. Several commercially available dental resin-based composites were chosen to ensure a representative sample that reflected variations in monomer composition, filler content, and polymerization kinetics. The selected dental resin-based composites (monomers and fillers) for this study are shown in Table 1.

**Table 1:** Components and chemical structures of selected dental resin-based composites

Component	Chemical Structure	Density (g/cm³)
Monomers		
2-Hydroxyethyl methacrylate (HEMA)		1.03
Bisphenol A glycidyl methacrylate (Bis-GMA)		1.16
Ethoxylated bisphenol A dimethacrylate (EBPADMA)		1.12
Triethylene glycol dimethacrylate (TEGDMA)		1.07
Urethane dimethacrylate (UDMA)		1.11
**Fillers**		
Calcium Carbonate (CaCO_3_)		2.80
Cerium(IV) Oxide (CeO_2_)		7.13
Hydroxyapatite (HCa_5_O_13_P_3_)		3.16
Silica particles (SiO_2_)		2.58
Silicon Nitride (Si_3_N_4_)		3.30

The rationale behind this selection is rooted in the need to capture a broad spectrum of materials used in clinical practice. This approach aligned with previous studies emphasizing material composition’s significance in determining dental composites’ mechanical and adhesive properties [1]. By studying a diverse set of materials, this study aimed to provide insights that could be broadly applicable to clinical scenarios.

### Selection of potential modifiers

The research proceeded with identifying candidate modifiers that could enhance dental resin composites’ adhesion and biomechanical properties. These modifiers encompassed a range of substances, including adhesion promoters, reinforcing agents, and other additives known for their potential impact on composite properties (Table 2).

**Table 2:** Coupling agents in dental resin-based composites

Component	Chemical Structure	Density (g/cm³)
Coupling Agents		
3-Methacryloxypropyltriethoxysilane (3-MPTS)		1.04
3-(Trimethoxysilyl)propyl methacrylate (TRIS)		0.93
Isocyanatopropyltriethoxysilane (ICPTES)		0.99
N-(2-Aminoethyl)-3-aminopropyltrimethoxysilane (AEAPTMS)		0.97
Vinyltriethoxysilane (VTES)		0.90

An extensive review of the existing literature and experimental data on dental materials guided the selection of these modifiers. Previous studies have explored various modifiers, such as silanes and coupling agents, that could significantly influence adhesion [29, 30]. Identifying these modifiers was crucial, as they formed the basis for subsequent simulations and experimentation.

### Molecular modelling of resin-based composite components

This section elaborates on the critical steps in generating precise three-dimensional (3D) molecular structures for the fundamental elements of dental resin-based composites, encompassing monomers, fillers, and coupling agents. These computational models form the bedrock upon which we gain a comprehensive understanding of the complex molecular interactions that dictate the behavior of these composites. This process was initiated by procuring accurate 3D representations of the core components of the composite. To obtain these 3D structures in the structure data format (SDF), ligands consisting of monomers, fillers, and coupling agents were sourced from the PubChem database (https://pubchem.ncbi.nlm.nih.gov/). Subsequently, each ligand was subjected to a crucial minimization process using OpenBabel version 3.0.1. This step was essential to ensure that the molecular structures were energetically stable and conformed to the most favorable spatial arrangements. Using the PubChem database and OpenBabel version 3.0.1, we guaranteed that our computational models accurately represented the real-world molecular structures of the composite constituents.

- Monomers: Monomers, the primary building blocks of the resin matrix, play a pivotal role in determining the properties of the composite. In this step, the molecular structures of selected monomers, including HEMA, Bis-GMA, EBPADMA, TEGDMA, and UDMA, were constructed. This process involved defining the bond lengths, bond angles, and dihedral angles to replicate the conformation of these monomers in three dimensions faithfully.
- Fillers: Fillers, including calcium carbonate, cerium(IV) oxide, hydroxyapatite, silica particles, and silicon nitride, significantly influence the mechanical properties of composites. The 3D structures of these fillers were meticulously created using ChemDraw Ultra 12.0 (PerkinElmer Inc.) [31] to capture their crystalline or amorphous nature. This endeavor was crucial for understanding how the fillers were dispersed within the resin matrix and how their surface properties contributed to the adhesion and biomechanical performance.
- Coupling Agents: Coupling agents, such as 3-MPTS, TRIS, ICPTES, AEAPTMS, and VTES, enhanced the adhesion between the organic and inorganic components. ChemDraw Ultra and OpenBabel enabled the construction of 3D structures of these coupling agents, focusing on their unique functional groups that facilitated bonding with organic monomers and inorganic fillers.

### Molecular docking simulations

Molecular docking simulations were executed using the HADDOCK stand-alone version. The HADDOCK software provides a versatile platform for these simulations, offering robust algorithms for exploring binding modes and energetics [32]. The key objective was to predict and elucidate the interactions between the components of dental resin-based composites (monomers, fillers, and coupling agents). Importantly, in this step, the simulations were carried out without including specific receptors, which allowed for a more comprehensive and open-ended exploration of the intermolecular interactions. Active residues for each ligand were meticulously specified to delineate crucial interaction sites, whereas passive residues, representing additional contributing elements, were also defined. Ambiguous Interaction Restraints (AIRs) were incorporated to guide the docking process, notably when experimental data hinted at the approximate binding sites. Distance restraints of 1.0 Å were employed to enforce specific geometrical relationships between atoms or residues of the two ligands. Flexible docking was facilitated by allowing conformational changes in the ligands during simulation. Subsequently, the generated docking solutions were subjected to cluster analysis to identify the representative and stable complexes. Energy scoring was crucial for evaluating the favorability of binding, considering the various scoring functions available in HADDOCK.

### Molecular dynamics (MD) simulations

This section provides a detailed account of MD simulations, a critical component of the research methodology aimed at understanding the behavior of dental resin-based composite materials over time and the evolution of intermolecular interactions under dynamic conditions. Molecular dynamics simulations are indispensable tools for investigating the dynamic behavior of dental resin-based composites over extended periods. The MD simulation focused on the biomechanical properties of the dental composite complexes (Young’s modulus, shear modulus, and flexural strength). Hydrogen bonds (as stabilizers of ligand-ligand complex interactions) were also calculated. The Forcite module with the COMPASS II force field was used in the simulation to represent intra- and intermolecular interactions. This module and force field are ideal for simulations of polymers and inorganic compounds [33]. In the initial stage of the MD simulation, the energy of all dental composite complexes (the result of docking simulation) was minimized with a force and energy convergence of 0.001 kcal/mol/A˚ and 5 × 10^−5^ kcal/mol, respectively. Subsequently, the system underwent a controlled simulation of the number of particles, system volume, and temperature (NVT) ensemble for 50 ns, manipulating the particle number, system volume, and temperature. Another 50 ns simulation followed this in the number of particles, system pressure, and temperature (NPT) ensemble, regulating particle number, system pressure, and temperature at 1.0 bar and 298 K. Pressure and temperature control were facilitated through the Nose-Hoover Thermostat-Langevin and Berendsen barostats, each with damping constants of 0.1 and 1.0 ns, respectively. Subsequently, the dental composite underwent additional equilibration in the NVT ensemble at 298 K for 50 ns. The Verlet velocity integration algorithm was employed with a time step of 1.0 ns, and a high calculation quality was chosen to enhance computational accuracy. Van der Waals and electrostatic interactions were computed using the particle mesh Ewald method [34]. The resulting equilibrium molecular structure of the dental resin composite was used for subsequent structural and mechanical analyses.

## Results

### Molecular docking simulations for possible combinations of monomers, fillers, and coupling agents

Molecular docking simulations were employed to investigate the potential interactions between the core components of dental resin-based composites: monomers, fillers, and coupling agents. The primary objective of this simulation was to assess the binding energy, van der Waals energy, electrostatic energy, and desolvation energy for various combinations of these components. These results provide crucial insights into the stability and affinity of these combinations, which are central to the design and performance of dental composites. Table 3 presents the results of molecular docking simulations for various combinations of monomers, fillers, and coupling agents. The binding energy values and different energy components were reported in kilocalories per mole (kcal/mol) and were determined with precision. We observed notable variations in the binding energies across different combinations, signifying diverse interactions between the core components. For instance, combinations involving specific monomers exhibit more favorable binding energies than others, suggesting their potential as better candidates for dental resin-based composites.

**Table 3:** Molecular docking simulation results for possible combinations of monomers, fillers, and coupling agents in dental resin-based composites. The gray box indicates the most favorable combination

Monomer	Filler	Coupling Agent	Binding energy (kcal/mol)	Van der Waals energy	Electrostatic energy	Desolvation energy
HEMA	CaCO_3_	3-MPTS	-4.8 ± 0.2	-3.6 ± 0.1	-23.6 ± 1.0	1.2 ± 0.1
		TRIS	-4.6 ± 0.3	-3.2 ± 0.1	-27.8 ± 0.6	1.5 ± 0.3
		ICPTES	-5.2 ± 0.3	-3.6 ± 0.2	-25.1 ± 2.5	0.9 ± 0.1
		AEAPTMS	-4.7 ± 0.3	-3.2 ± 0.2	-27.2 ± 0.3	1.2 ± 0.2
		VTES	-12.7 ± 0.0	-8.4 ± 0.0	-38.5 ± 0.0	-0.5 ± 0.0
	CeO_2_	3-MPTS	-2.4 ± 0.0	-2.6 ± 0.0	-3.9 ± 0.5	0.5 ± 0.1
		TRIS	-2.2 ± 0.1	-2.8 ± 0.0	0.7 ± 1.6	0.5 ± 0.0
		ICPTES	-2.3 ± 0.2	-2.8 ± 0.1	-1.0 ± 0.4	0.6 ± 0.2
		AEAPTMS	-1.6 ± 0.3	-2.0 ± 0.4	1.4 ± 0.1	0.2 ± 0.1
		VTES	-3.3 ± 0.4	-3.8 ± 0.0	-10.4 ± 0.4	1.6 ± 0.5
	HCa_5_O_13_P_3_	3-MPTS	-2.6 ± 0.1	-3.6 ± 0.1	-10.6 ± 2.6	2.1 ± 0.0
		TRIS	-3.1 ± 0.1	-3.1 ± 0.2	-19.7 ± 1.6	2.0 ± 0.2
		ICPTES	-3.6 ± 0.0	-3.5 ± 0.1	-20.0 ± 0.5	1.9 ± 0.1
		AEAPTMS	-3.1 ± 0.0	-3.8 ± 0.2	-16.1 ± 1.3	2.3 ± 0.1
		VTES	-10.0 ± 0.0	-6.8 ± 0.0	-33.3 ± 0.0	0.1 ± 0.0
	SiO_2_	3-MPTS	-14.1 ± 0.0	-9.3 ± 0.0	-31.9 ± 0.0	-1.5 ± 0.0
		TRIS	-21.1 ± 0.0	-9.7 ± 0.0	-92.0 ± 0.0	-2.2 ± 0.0
		ICPTES	-13.1 ± 0.0	-7.8 ± 0.1	-44.4 ± 0.1	-0.9 ± 0.0
		AEAPTMS	-15.2 ± 0.0	-8.8 ± 0.0	-50.0 ± 0.0	-1.3 ± 0.0
		VTES	-10.1 ± 0.0	-6.2 ± 0.0	-10.5 ± 0.0	-2.8 ± 0.0
	Si_3_N_4_	3-MPTS	-9.8 ± 1.3	0.1 ± 1.4	-45.8 ± 7.7	-0.7 ± 0.7
		TRIS	-10.2 ± 0.0	-5.4 ± 0.0	-55.4 ± 0.0	0.7 ± 0.0
		ICPTES	-10.7 ± 0.0	-4.9 ± 0.0	-67.6 ± 0.0	0.9 ± 0.0
		AEAPTMS	-12.8 ± 0.0	-6.0 ± 0.0	-65.7 ± 0.0	-0.2 ± 0.0
		VTES	-8.8 ± 0.3	-3.3 ± 0.0	-51.8 ± 4.2	-0.3 ± 0.2
Bis-GMA	CaCO_3_	3-MPTS	-9.7 ± 0.5	-5.6 ± 0.4	-55.1 ± 3.9	1.4 ± 0.3
		TRIS	-9.8 ± 0.3	-5.7 ± 0.6	-59.1 ± 2.6	1.8 ± 0.2
		ICPTES	-10.4 ± 0.2	-6.1 ± 0.3	-58.5 ± 1.4	1.6 ± 0.2
		AEAPTMS	-9.9 ± 0.4	-5.9 ± 0.3	-56.8 ± 1.7	1.7 ± 0.2
		VTES	-21.6 ± 1.2	-13.2 ± 0.8	-32.4 ± 0.8	-5.2 ± 0.3
	CeO_2_	3-MPTS	-4.7 ± 0.2	-5.1 ± 0.3	0.0 ± 1.7	0.4 ± 0.3
		TRIS	-5.0 ± 0.1	-5.2 ± 0.3	0.4 ± 1.3	0.2 ± 0.3
		ICPTES	-4.9 ± 0.6	-5.1 ± 0.2	0.6 ± 0.6	0.1 ± 0.5
		AEAPTMS	-4.9 ± 0.1	-5.0 ± 0.2	1.1 ± 0.7	0.0 ± 0.0
		VTES	-22.6 ± 0.0	-16.7 ± 0.0	-8.2 ± 0.0	-5.1 ± 0.0
	HCa_5_O_13_P_3_	3-MPTS	-6.9 ± 0.2	-6.0 ± 0.3	-40.2 ± 3.1	3.1 ± 0.4
		TRIS	-7.8 ± 0.7	-6.7 ± 0.3	-39.8 ± 0.6	2.9 ± 0.3
		ICPTES	-8.0 ± 0.5	-6.4 ± 0.5	-42.7 ± 1.0	2.7 ± 0.1
		AEAPTMS	-6.9 ± 0.3	-5.6 ± 0.1	-40.7 ± 0.0	2.7 ± 0.4
		VTES	-34.8 ± 0.0	-26.1 ± 0.0	-48.5 ± 0.0	-3.9 ± 0.0
	SiO_2_	3-MPTS	-30.9 ± 0.0	-19.6 ± 0.0	-37.4 ± 0.0	-7.5 ± 0.0
		TRIS	-35.8 ± 0.0	-21.5 ± 0.0	-45.4 ± 0.0	-9.7 ± 0.0
		ICPTES	-30.1 ± 0.0	-17.9 ± 0.0	-70.0 ± 0.0	-5.2 ± 0.0
		AEAPTMS	-32.4 ± 0.0	-20.7 ± 0.0	-40.8 ± 0.0	-7.6 ± 0.0
		VTES	-29.7 ± 0.0	-18.9 ± 0.0	-20.8 ± 0.0	-8.7 ± 0.0
	Si_3_N_4_	3-MPTS	-12.6 ± 0.0	-6.0 ± 0.0	-49.3 ± 0.0	-1.7 ± 0.0
		TRIS	-13.7 ± 0.0	-5.4 ± 0.0	-74.1 ± 0.0	-0.9 ± 0.0
		ICPTES	-13.6 ± 0.0	-2.5 ± 0.0	-105.7 ± 0.0	-0.5 ± 0.0
		AEAPTMS	-13.1 ± 0.0	-2.7 ± 0.0	-85.5 ± 0.0	-1.8 ± 0.0
		VTES	-24.7 ± 0.0	-12.2 ± 0.0	-61.0 ± 0.0	-6.5 ± 0.0
EBPADMA	CaCO_3_	3-MPTS	-7.9 ± 0.0	-4.3 ± 0.1	-45.4 ± 0.7	1.0 ± 0.0
		TRIS	-8.3 ± 0.4	-6.2 ± 0.2	-36.6 ± 0.9	1.5 ± 0.7
		ICPTES	-7.2 ± 1.1	-4.8 ± 0.4	-40.7 ± 3.1	1.6 ± 0.4
		AEAPTMS	-8.2 ± 0.7	-5.9 ± 0.0	-37.2 ± 4.0	1.5 ± 0.3
		VTES	-29.4 ± 0.1	-22.3 ± 0.2	-32.7 ± 3.5	-3.9 ± 0.2
	CeO_2_	3-MPTS	-4.1 ± 0.4	-4.7 ± 0.3	-0.9 ± 0.9	0.7 ± 0.3
		TRIS	-4.5 ± 0.4	-5.0 ± 0.3	-0.0 ± 1.5	0.5 ± 0.4
		ICPTES	-5.0 ± 0.1	-5.3 ± 0.2	-0.8 ± 0.3	0.4 ± 0.2
		AEAPTMS	-5.0 ± 0.2	-5.4 ± 0.1	0.2 ± 1.2	0.4 ± 0.3
		VTES	-26.7 ± 0.0	-21.5 ± 0.0	-18.8 ± 0.0	-3.3 ± 0.0
	HCa_5_O_13_P_3_	3-MPTS	-6.1 ± 0.9	-5.8 ± 0.4	-26.9 ± 2.6	2.3 ± 0.2
		TRIS	-6.9 ± 0.0	-6.2 ± 0.0	-28.7 ± 0.0	2.2 ± 0.0
		ICPTES	-7.3 ± 0.0	-7.0 ± 0.0	-30.0 ± 0.0	2.7 ± 0.0
		AEAPTMS	-6.0 ± 0.4	-6.0 ± 0.1	-26.9 ± 1.0	2.7 ± 0.2
		VTES	-25.6 ± 0.0	-20.3 ± 0.0	-37.7 ± 0.0	-1.6 ± 0.0
	SiO_2_	3-MPTS	-26.3 ± 0.0	-16.2 ± 0.0	-39.0 ± 0.0	-6.2 ± 0.0
		TRIS	-30.5 ± 0.0	-13.9 ± 0.0	-98.2 ± 0.0	-6.8 ± 0.0
		ICPTES	-27.0 ± 0.0	-15.9 ± 0.0	-66.8 ± 0.0	-4.4 ± 0.0
		AEAPTMS	-29.2 ± 0.0	-15.1 ± 0.0	-104.8 ± 0.0	-3.6 ± 0.0
		VTES	-30.3 ± 0.0	-21.0 ± 0.0	-4.7 ± 0.0	-8.9 ± 0.0
	Si_3_N_4_	3-MPTS	-16.2 ± 0.0	-6.8 ± 0.0	-78.4 ± 0.0	-1.6 ± 0.0
		TRIS	-14.4 ± 0.0	-6.8 ± 0.0	-70.4 ± 0.0	-0.5 ± 0.0
		ICPTES	-12.9 ± 0.0	-3.7 ± 0.0	-74.9 ± 0.0	-1.7 ± 0.0
		AEAPTMS	-13.5 ± 0.0	-3.0 ± 0.0	-100.8 ± 0.0	-0.4 ± 0.0
		VTES	-24.9 ± 0.0	-13.2 ± 0.0	-72.0 ± 0.0	-4.5 ± 0.0
TEGDMA	CaCO_3_	3-MPTS	-8.7 ± 0.3	-3.5 ± 0.1	-67.2 ± 3.5	1.5 ± 0.3
		TRIS	-9.1 ± 0.2	-3.3 ± 0.1	-70.9 ± 1.2	1.3 ± 0.0
		ICPTES	-9.5 ± 0.7	-3.6 ± 0.1	-71.6 ± 1.6	1.2 ± 0.5
		AEAPTMS	-9.1 ± 0.3	-3.7 ± 0.1	-69.1 ± 1.8	1.4 ± 0.1
		VTES	-20.8 ± 0.0	-12.0 ± 0.0	-65.8 ± 0.0	-2.2 ± 0.0
	CeO_2_	3-MPTS	-2.6 ± 0.1	-3.2 ± 0.0	2.0 ± 0.0	0.4 ± 0.1
		TRIS	-2.6 ± 0.3	-3.2 ± 0.1	-0.4 ± 0.4	0.6 ± 0.2
		ICPTES	-2.4 ± 0.4	-2.9 ± 0.3	-4.6 ± 0.2	0.9 ± 0.1
		AEAPTMS	-2.6 ± 0.3	-3.2 ± 0.2	0.9 ± 0.9	0.5 ± 0.2
		VTES	-15.0 ± 0.0	-10.2 ± 0.0	-31.8 ± 0.0	-1.6 ± 0.0
	HCa_5_O_13_P_3_	3-MPTS	-7.5 ± 0.0	-3.7 ± 0.1	-57.5 ± 1.8	1.9 ± 0.2
		TRIS	-6.3 ± 0.3	-4.2 ± 0.2	-48.3 ± 0.2	2.8 ± 0.1
		ICPTES	-7.1 ± 0.1	-3.6 ± 0.0	-55.2 ± 0.6	2.0 ± 0.0
		AEAPTMS	-7.4 ± 0.0	-3.2 ± 0.1	-61.1 ± 1.5	1.9 ± 0.0
		VTES	-17.8 ± 0.0	-12.2 ± 0.0	-46.8 ± 0.0	-0.9 ± 0.0
	SiO_2_	3-MPTS	-22.6 ± 0.0	-11.9 ± 0.0	-69.9 ± 0.0	-3.6 ± 0.0
		TRIS	-37.6 ± 0.0	-14.6 ± 0.0	-184.4 ± 0.0	-4.5 ± 0.0
		ICPTES	-22.8 ± 0.0	-10.1 ± 0.0	-104.8 ± 0.0	-2.2 ± 0.0
		AEAPTMS	-23.2 ± 0.0	-8.9 ± 0.0	-123.5 ± 0.0	-1.9 ± 0.0
		VTES	-19.5 ± 0.0	-12.4 ± 0.0	-45.5 ± 0.0	-2.5 ± 0.0
	Si_3_N_4_	3-MPTS	-9.0 ± 0.6	-4.7 ± 0.6	-56.1 ± 2.5	1.3 ± 0.3
		TRIS	-12.2 ± 0.1	-3.8 ± 0.1	-94.1 ± 4.2	1.0 ± 0.3
		ICPTES	-13.6 ± 0.0	-3.2 ± 0.0	-97.2 ± 0.0	-0.7 ± 0.0
		AEAPTMS	-14.0 ± 0.0	-3.8 ± 0.0	-84.0 ± 0.0	-1.8 ± 0.0
		VTES	-19.5 ± 0.0	-9.2 ± 0.0	-70.4 ± 0.0	-3.3 ± 0.0
UDMA	CaCO_3_	3-MPTS	-11.3 ± 0.6	-5.2 ± 0.1	-77.3 ± 4.0	1.7 ± 0.2
		TRIS	-10.9 ± 0.5	-4.7 ± 0.3	-77.8 ± 3.0	1.6 ± 0.2
		ICPTES	-11.1 ± 0.6	-5.0 ± 0.3	-76.2 ± 4.1	1.5 ± 0.2
		AEAPTMS	-11.0 ± 0.5	-5.1 ± 0.2	-74.7 ± 3.5	1.6 ± 0.1
		VTES	-14.1 ± 0.5	-8.4 ± 0.8	-56.3 ± 2.6	-0.1 ± 0.0
	CeO_2_	3-MPTS	-3.6 ± 0.4	-3.7 ± 0.2	-0.1 ± 0.3	0.1 ± 0.1
		TRIS	-3.5 ± 0.4	-4.2 ± 0.2	1.1 ± 0.6	0.5 ± 0.3
		ICPTES	-4.0 ± 0.3	-4.2 ± 0.1	1.2 ± 0.9	0.1 ± 0.3
		AEAPTMS	-3.6 ± 0.5	-3.8 ± 0.4	1.1 ± 0.5	0.1 ± 0.1
		VTES	-21.8 ± 0.0	-16.0 ± 0.0	-24.6 ± 0.0	-3.4 ± 0.0
	HCa_5_O_13_P_3_	3-MPTS	-9.4 ± 0.0	-4.6 ± 0.1	-71.8 ± 1.8	2.3 ± 0.2
		TRIS	-5.5 ± 0.3	-3.6 ± 0.2	-42.0 ± 0.2	2.4 ± 0.1
		ICPTES	-8.6 ± 0.1	-4.3 ± 0.0	-66.7 ± 0.6	2.4 ± 0.0
		AEAPTMS	-8.9 ± 0.0	-3.8 ± 0.1	-73.9 ± 1.5	2.3 ± 0.0
		VTES	-28.3 ± 0.0	-5.8 ± 0.1	-91.4 ± 1.8	3.0 ± 0.2
	SiO_2_	3-MPTS	-28.4 ± 0.0	-15.1 ± 0.0	-68.4 ± 0.0	-6.5 ± 0.0
		TRIS	-33.0 ± 0.0	-15.7 ± 0.0	-99.8 ± 0.0	-7.3 ± 0.0
		ICPTES	-27.6 ± 0.0	-14.7 ± 0.0	-57.5 ± 0.0	-7.1 ± 0.0
		AEAPTMS	-28.2 ± 0.0	-14.3 ± 0.0	-68.6 ± 0.0	-7.1 ± 0.0
		VTES	-31.1 ± 0.0	-20.0 ± 0.0	-30.0 ± 0.0	-8.1 ± 0.0
	Si_3_N_4_	3-MPTS	-12.0 ± 0.7	-6.2 ± 0.2	-83.2 ± 7.2	2.5 ± 0.3
		TRIS	-14.2 ± 0.0	-4.1 ± 0.0	-95.3 ± 0.0	-0.6 ± 0.0
		ICPTES	-14.1 ± 0.0	-3.1 ± 0.0	-97.4 ± 0.0	-1.2 ± 0.0
		AEAPTMS	-13.9 ± 0.6	-7.3 ± 0.4	-83.3 ± 8.3	1.7 ± 0.1
		VTES	-28.7 ± 0.0	-14.3 ± 0.8	-98.7 ± 4.2	-4.6 ± 0.3

**Fig. 1:**
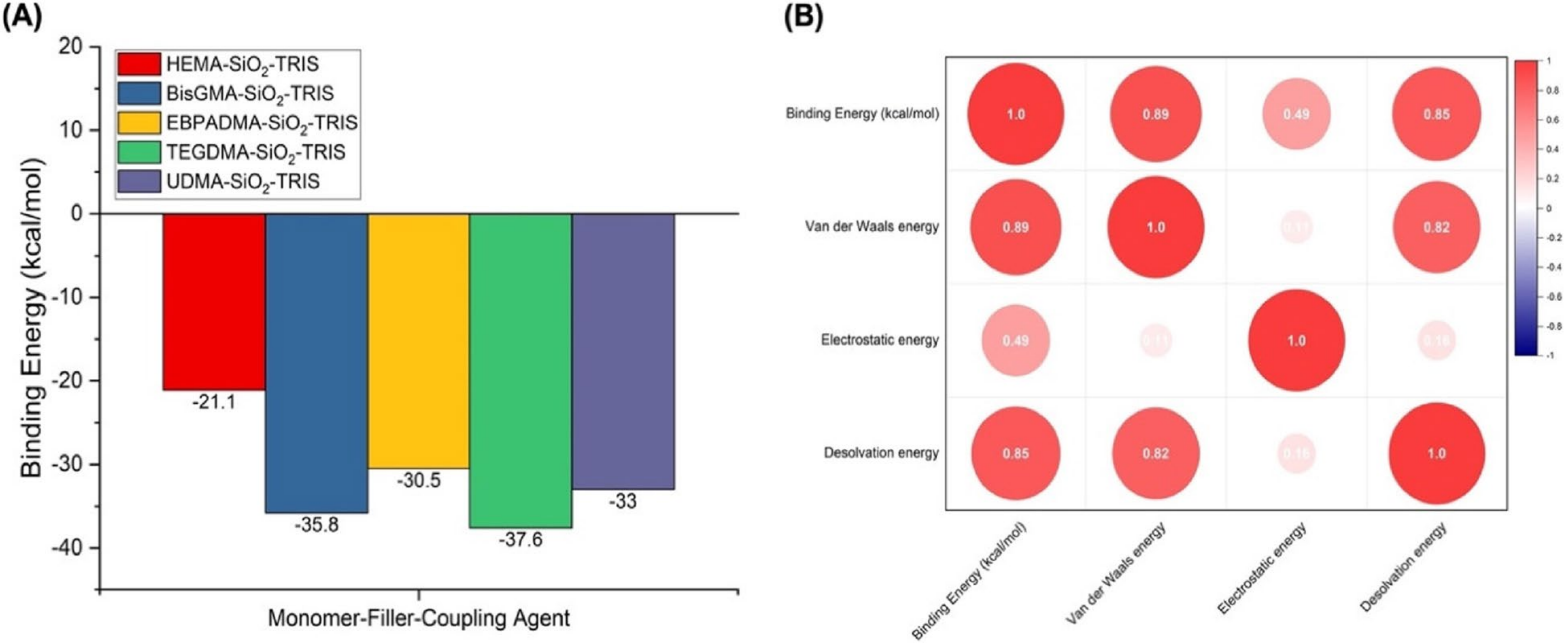
Results of the molecular docking screening simulation. **A** The best monomer-filler-coupling agent combination for each monomer type with the lowest binding energy value related to the adhesion strength. **B** Correlation matrix of binding energy (kcal/mol) with individual energy components. Figure 1b provides a correlation matrix that quantifies the degree of association between the binding energy, van der Waals energy, electrostatic energy, and desolvation energy. The values in the matrix range from − 1 to 1, where 1 signifies a perfect positive correlation, -1 indicates a perfect negative correlation, and 0 represents no correlation. Binding energy, as the central parameter in our study, exhibited notable correlations with other energy components. The negative sign indicates that energy is released during the formation of the complex, making it thermodynamically favorable [38]. Therefore, when comparing different combinations or complexes, a higher (more negative) binding energy corresponds to a higher adhesion strength. Combinations featuring specific monomers, fillers, and coupling agents demonstrated more favorable binding energies than the others. This observation suggests that these monomers are promising candidates for enhancing the adhesive properties of dental resin-based composites

Monomers, including HEMA, Bis-GMA, EBPADMA, TEGDMA, and UDMA, were assessed for their interactions with different fillers and coupling agents. The calculated binding energies revealed distinct patterns. HEMA exhibited a strong interaction with SiO_2_ across various fillers, with a remarkable binding energy of -21.1 kcal/mol when coupled with TRIS, indicating a favorable adhesion profile. This behavior may be attributed to the electrostatic energy component, which is significantly attractive in the presence of TRIS. For Bis-GMA, the results also indicated substantial interactions with TRIS. The highest binding energy observed for Bis-GMA was − 35.8 kcal/mol, also with SiO_2_. The interplay between the van der Waals and electrostatic energies governed these interactions. EBPADMA displayed notable interactions with TRIS when paired with SiO_2_, resulting in a binding energy of -30.5 kcal/mol. The contribution of the electrostatic energy was substantial. Conversely, EBPADMA demonstrated relatively low binding affinities with other coupling agents, such as 3-MPTS and ICPTES, across various fillers. The weaker interactions might be attributed to the balance between the van der Waals and electrostatic energies. TEGDMA’s interactions were consistently influenced by TRIS, with binding energies ranging from − 2.6 to -37.6 kcal/mol. TEGDMA, when combined with SiO_2_ and TRIS, exhibited a binding energy of -37.6 kcal/mol. A remarkable aspect of these interactions is the role of electrostatic energy, which is more pronounced than van der Waals energy. UDMA generally exhibits moderate binding affinities when interacting with various fillers and coupling agents. The most robust interactions were observed with TRIS, emphasizing a binding energy of -33.0 kcal/mol for UDMA paired with SiO_2_. Both the van der Waals and electrostatic components played integral roles in these interactions. The best monomer-filler-coupling agent combination for each monomer type with the lowest binding energy is depicted in Fig. 1a, where TEGDMA-SiO_2_-TRIS possessed the lowest binding energy (37.6 kcal/mol). Interestingly, SiO_2_ as a filler and TRIS as a coupling agent significantly influenced the binding energies across all monomers. SiO_2_ is known for its large surface area and can provide a large contact area for monomer interactions [35]. TRIS, a tris(hydroxymethyl)aminomethane, is commonly used as a coupling agent in polymer composite formulations owing to its hydroxyl and amine functional groups, which can participate in various interactions with monomers [36, 37]. The consistently favorable results of SiO_2_ and TRIS across multiple monomers suggest their versatility and effectiveness in promoting strong interactions, making them valuable components for developing composite materials for various dental applications. The binding energies obtained from the molecular docking simulations serve as crucial indicators of the adhesion strength of dental resin-based composites. In the context of this study, a higher magnitude of negative binding energy signifies stronger intermolecular interactions, highlighting superior adhesion properties—the more negative the binding energy, the more stable and favorable the binding between the components.

In terms of van der Waals energy, a strong positive correlation (*r* = 0.89) underscores its pivotal role in determining the binding energy. As the van der Waals energy increases, there is a corresponding increase in the overall binding energy. This underscores the significant role that van der Waals forces play in shaping the overall binding energy, as supported by several studies [39–41]. In contrast, a positive correlation of 0.49 was evident when considering electrostatic energy, albeit weaker than the correlation observed with van der Waals energy. This observation suggests that while electrostatic interactions contribute to the binding energy, their influence is not dominant, and other contributing factors may come into play, as demonstrated in previous research [42, 43]. Examining desolvation energy, a substantial positive correlation of 0.85 was detected, emphasizing the significant impact of desolvation energy on binding energy [44, 45]. This correlation suggests a strong connection between the ability to desolvate or displace solvent molecules from the binding site and the overall binding energy. This correlation indicates that as the desolvation energy increases, there is a corresponding increase in the binding energy. This finding is particularly relevant to adhesion in the context of dental resin-based composites. The desolvation energy plays a crucial role in the adhesion process by influencing the interactions between different composite material components. When solvent molecules are effectively displaced from the binding site, they enhance the interaction and binding strength between the components, contributing to a more stable complex. A higher desolvation energy is associated with a greater ability to remove solvent molecules, leading to thermodynamically favorable and more robust binding interactions. Therefore, the positive correlation observed in this study underscores the importance of desolvation energy in influencing the adhesion properties of dental resin-based composites.

### Molecular dynamics simulations for the best combinations of monomers, fillers, and coupling agents

To delve deeper into the impact of fillers and coupling agents on the monomer within dental composites, micromechanics models have been introduced to quantitatively predict Young’s modulus, Shear Modulus, and flexural strength of the generated dental composite based on the MD simulation.

1. Young’s modulus (E)

Young’s modulus is often estimated using the rule of mixtures, especially for composite materials.

1 $$E= {\sum }_{i=1}^{n}{f}_{i} \cdot {E}_{i}$$

Where:

- $E$ is the Young’s modulus of the composite.
- ${f}_{i}$is the volume fraction of component $i$.
- ${E}_{i}$is the Young’s modulus of component $i$.

2. Shear modulus (G)

The shear modulus can also be estimated using the rule of mixtures.

2 $$G=\frac{1}{2} {\sum }_{i=1}^{n}{f}_{i} \cdot{G}_{i}$$

Where:

- $G$ is the shear modulus of the composite.
- ${f}_{i}$ is the volume fraction of component $i$.
- ${G}_{i}$ is the Shear modulus of component $i$.

3. Flexural strength

The flexural strength of a composite material can be estimated using the following equation:

3 $${\sigma }_{flexural}= \sqrt{{\sigma }_{tensile}\cdot {\sigma }_{compressive} }$$

Where:

- ${\sigma }_{flexural}$ is the flexural strength.
- ${\sigma }_{tensile}$ is the tensile strength.
- ${\sigma }_{compressive}$ is the compressive strength.

**Fig. 2:**
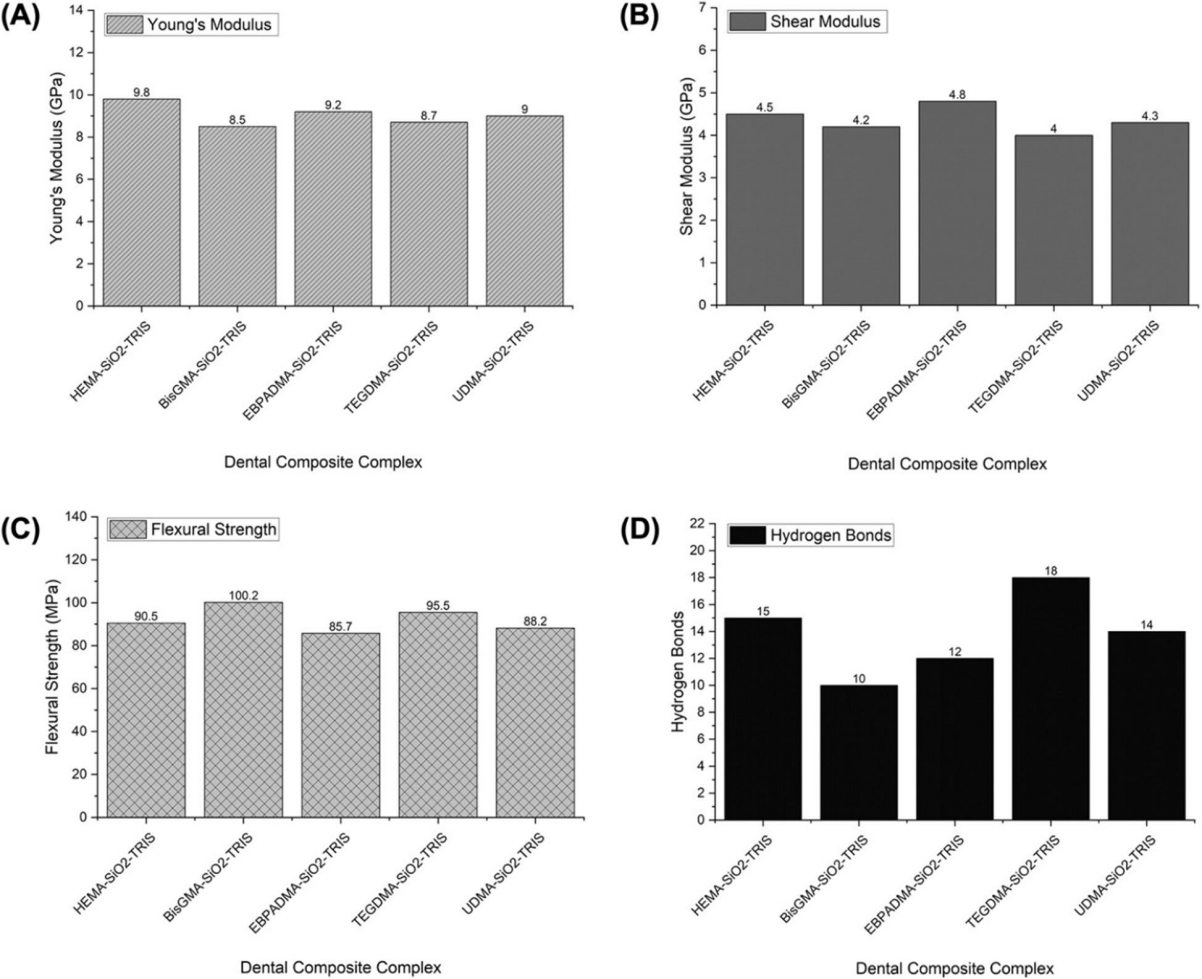
Molecular dynamics simulation results for the best dental composite complex. **A** Young’s modulus (GPa). **B** Shear modulus (GPa). **C** Binding strength (MPa). **D** Hydrogen bonds

Figure 2 presents the mechanical properties of various dental composite complexes, as determined through the MD simulation. These simulations offer a detailed atomic-level understanding of resin-based composites’ structural integrity and performance over time, allowing for valuable insights into their applicability in restorative dentistry. Young’s modulus, representing material stiffness, is crucial in dental applications [46]. Incorporating silica nanoparticles enhanced stiffness, supporting the notion that including inorganic fillers positively influences mechanical properties [47]. The stiffness of HEMA-SiO_2_-TRIS, as indicated by its high Young’s modulus of 9.8 GPa, underscores the reinforcing effect of silica nanoparticles and highlights its potential in load-bearing dental restorations. BisGMA-SiO_2_-TRIS and EBPADMA-SiO_2_-TRIS demonstrate competitive Young’s modulus values (8.5 GPa and 9.2 GPa, respectively), reinforcing the significance of the monomer composition in influencing mechanical properties. BisGMA, a monomer widely used in dental composites, has been associated with high stiffness, corroborating the findings of this study. The observed trends align with previous research emphasizing monomer selection’s impact on dental composites’ mechanical behavior [48].

The shear modulus, indicative of a material’s response to shear stress, is critical for assessing the stability of a complex under various loading conditions [49]. In this study, EBPADMA-SiO_2_-TRIS demonstrated the highest shear modulus at 4.8 GPa, indicating robust resistance to shear forces. HEMA-SiO_2_-TRIS and UDMA-SiO_2_-TRIS also exhibited notable shear moduli, highlighting their ability to withstand shear stress. On the other hand, flexural strength, indicative of a material’s resistance to bending stresses, is a crucial parameter in dental restorations [50]. BisGMA-SiO_2_-TRIS displays the highest flexural strength at 100.2 MPa, indicating excellent resistance to bending forces. TEGDMA-SiO_2_-TRIS and HEMA-SiO_2_-TRIS also exhibited substantial flexural strength, suggesting their suitability for applications requiring resistance to bending stresses. Furthermore, the number of hydrogen bonds plays a pivotal role in determining the structural stability of dental composites. The dental composite TEGDMA-SiO_2_-TRIS, characterized by the highest number of hydrogen bonds (18), suggests solid intermolecular interactions. This is likely to contribute to the increased stiffness and resistance to deformation, as reflected in the high values of Young’s modulus and shear modulus. The hydrogen bonds act as additional molecular connections, reinforcing the structural integrity of the composite. Dental composites such as HEMA-SiO_2_-TRIS and UDMA-SiO_2_-TRIS, which exhibit a considerable number of hydrogen bonds, also exhibit good structural stability. This moderate level of hydrogen bonding likely contributes to balanced mechanical properties, with Young’s modulus and Shear’s modulus falling within a favorable range. The complete results are shown in Table 4.

**Table 4:** Mechanical properties of dental composite complexes based on MD simulation

Dental Composite Complex	Young's Modulus (GPa)	Shear Modulus (GPa)	Flexural Strength (MPa)	Hydrogen Bonds
HEMA-SiO_2_-TRIS	9.8	4.5	90.5	15
BisGMA-SiO_2_-TRIS	8.5	4.2	100.2	10
EBPADMA-SiO_2_-TRIS	9.2	4.8	85.7	12
TEGDMA-SiO_2_-TRIS	8.7	4.0	95.5	18
UDMA-SiO_2_-TRIS	9.0	4.3	88.2	14

Determining the “best” dental composite complex is a multifaceted decision that requires careful consideration of specific applications and their corresponding material requirements. From the perspective of adhesion, the consistent augmentation of the adhesion power through SiO_2_ and TRIS is noteworthy, as evidenced by their robust binding energies. These components have emerged as key contributors to the adhesive properties of dental resin-based composites, laying a solid foundation for their efficacy in diverse dental applications.

Turning attention to biomechanical properties, the nuances of each composite complex outlined in Table 4 underscore the importance of aligning the choice with the intended use and sought-after material properties. In scenarios where stiffness is paramount, HEMA-SiO_2_-TRIS presents itself as a compelling option, given its high Young’s modulus. This composite complex may find practical applications when structural rigidity is critical, such as in restorations subjected to significant chewing stress. Conversely, for applications where resistance to bending stresses holds prominence, BisGMA-SiO_2_-TRIS has the highest flexural strength. This composite complex is particularly well suited for situations where the ability to withstand deformation is a critical mechanical requirement, ensuring the longevity and durability of dental restorations, especially in areas prone to bending stresses. Notably, EBPADMA-SiO_2_-TRIS and TEGDMA-SiO_2_-TRIS exhibited balanced mechanical properties, rendering them versatile options suitable for various applications. The equilibrium between stiffness, shear modulus, and bending strength positions these composite complexes as adaptable solutions capable of addressing a spectrum of clinical requirements. This versatility enhances their utility across different scenarios, offering a practical and well-rounded approach to dental composite selection.

## Discussion

### Biomechanical property enhancement through molecular docking and dynamics simulations

The amalgamation of dental materials has been demonstrated to significantly improve biocompatibility and functionality in various dental procedures. Notably, mineral trioxide aggregate (MTA) stands out as a prime example, renowned for its exceptional biocompatibility with oral and dental tissues. Initially engineered for dental root repair in endodontic interventions, MTA comprises commercial Portland cement fortified with bismuth oxide powder to enhance radiopacity [51]. Building upon these findings, our aim is to investigate the potential benefits of combining various monomers, fillers, and coupling agents within dental resin-based composites. In this study, we employed molecular docking and dynamics simulations to enhance the biomechanical properties of dental resin-based composites. By scrutinizing the intricate molecular interactions between monomers, fillers, and coupling agents, we aimed to redefine the design principles underlying dental biomaterials. Molecular docking simulations served as the foundation for elucidating the binding affinities between composite components. Through meticulous exploration of binding energies, van der Waals forces, electrostatic interactions, and desolvation energies, we discerned the driving forces shaping composite stability. Notably, certain combinations, such as HEMA-SiO_2_-TRIS and BisGMA-SiO_2_-TRIS, exhibited robust binding energies, underscoring their potential for enhancing adhesion. Moving beyond static interactions, molecular dynamics simulations provided dynamic insights into composite behavior under varying loading conditions. By quantifying Young’s modulus, shear modulus, and flexural strength, we gained a nuanced understanding of composite mechanics. The EBPADMA-SiO_2_-TRIS composite emerged as a frontrunner, showcasing exceptional shear modulus and balanced mechanical properties, essential for withstanding shear and bending stresses in clinical settings. The integration of molecular docking and dynamics simulations enabled a holistic approach to biomaterial optimization. By correlating molecular interactions with mechanical performance, we identified composite formulations with superior biomechanical properties. This molecular-driven approach offers a paradigm shift in dental biomaterial design, promising enhanced longevity and performance in restorative dentistry.

### Strengths, limitations, and clinical considerations

#### Strengths

The utilization of molecular docking and dynamics-driven simulations in optimizing dental resin-based composites offers several notable strengths. Firstly, these computational techniques provide a detailed atomic-level understanding of the interactions between composite components. By simulating the behavior of monomers, fillers, and coupling agents at the molecular level, researchers can gain insights into the mechanisms underlying adhesive and biomechanical properties. This granular understanding enables the identification of candidate modifiers with the potential to enhance composite performance. Molecular docking simulations allow for the rapid screening of numerous combinations of composite components. By predicting binding energies and interaction patterns, researchers can prioritize the most promising candidate materials for further investigation [52, 53]. This approach streamlines the materials selection process, enabling researchers to focus resources on the most viable options. Furthermore, molecular dynamics simulations offer a dynamic perspective on composite behavior over time. By subjecting composite complexes to simulated environmental conditions, researchers can assess their mechanical properties under realistic loading scenarios [54, 55]. This dynamic understanding is crucial for predicting composite performance in the complex oral environment, where materials are subjected to a range of mechanical stresses. The integration of computational simulations with experimental validation holds significant promise for accelerating materials development in dentistry. By combining computational predictions with empirical data, researchers can iteratively refine composite formulations, leading to the development of optimized materials with enhanced properties.

#### Limitations

Despite their strengths, computational simulations also have inherent limitations that must be considered. One limitation is the reliance on computational models, which may not fully capture the complexity of real-world systems. Simplifications and assumptions made in modelling approaches can introduce inaccuracies and limitations in simulation results [56, 57]. Additionally, the accuracy of computational predictions depends on the quality of input parameters and force fields used in simulations [58]. Inaccurate force field parameters or incomplete molecular representations can lead to biased results and erroneous conclusions. Therefore, careful validation of simulation results against experimental data is essential to ensure their reliability. Furthermore, computational simulations require significant computational resources and expertise to perform. High-performance computing infrastructure and specialized software are needed to conduct molecular docking and dynamics simulations efficiently. Moreover, interpreting simulation results and translating them into actionable insights require expertise in materials science, dentistry, and computational chemistry.

#### Clinical considerations

The findings from computational simulations have important implications for clinical practice in restorative dentistry. Optimized dental resin-based composites with enhanced adhesive and biomechanical properties can offer several clinical advantages. Improved adhesion properties can help minimize microleakage and secondary caries, leading to more durable and long-lasting restorations [59, 60]. By enhancing the bond strength between the composite and tooth structure, optimized materials can reduce the risk of restoration failure and the need for costly retreatment. Enhanced biomechanical properties, such as increased stiffness and flexural strength, can improve the longevity and performance of dental restorations [61, 62]. Materials with higher mechanical strength can withstand chewing forces more effectively, reducing the risk of fracture and wear over time. This can lead to better clinical outcomes and increased patient satisfaction with dental restorations [63, 64]. Furthermore, the versatility of optimized composite materials allows for their use in a wide range of clinical applications. From simple fillings to more complex restorations, these materials can provide aesthetic appeal, biocompatibility, and mechanical reliability, meeting the diverse needs of patients and clinicians alike. However, it is essential to recognize that the translation of computational findings into clinical practice requires careful validation and regulatory approval. Experimental validation of composite performance in vitro and in vivo is necessary to confirm the predicted benefits and ensure the safety and efficacy of new materials. Additionally, regulatory bodies such as the Food and Drug Administration (FDA) play a crucial role in evaluating and approving dental materials for clinical use, ensuring that they meet stringent standards for safety and performance.

## Conclusion

Molecular docking simulations investigated the interactions between monomers, fillers, and coupling agents in dental resin-based composites. The results revealed diverse binding energies across different combinations, with specific monomers exhibiting more favorable interactions. Notably, SiO_2_ and TRIS consistently influenced the binding energies across multiple monomers, suggesting their versatility in promoting strong interactions related to adhesion strength. Molecular dynamics simulations further elucidated the mechanical properties of dental composite complexes, showcasing the impact of monomer composition on the biomechanical properties (Young's modulus, shear modulus, flexural strength, and hydrogen bonding. These findings underscore the importance of careful selection based on specific application needs, with HEMA-SiO_2_-TRIS excelling in stiffness, BisGMA-SiO_2_-TRIS in bending strength, and EBPADMA-SiO_2_-TRIS offering balanced mechanical properties.

## Future works

Experimental validation of the computational findings would strengthen the reliability of the results. Conducting laboratory tests to verify the predicted mechanical properties and efficacy of the suggested dental composite formulations would be crucial for translating computational insights into practical applications. For instance, the combination of EBPADMA (primary monomer), SiO_2_ (filler), and TRIS (coupling agent) can be formulated using appropriate ratios. The polymerization process can be performed by applying light, heat, or chemical initiators. Subsequently, the adhesion and biomechanical properties were assessed through laboratory tests. Adhesion testing involves applying the composite to dental substrates and conducting micro-tensile or micro-shear bond strength tests using a universal testing machine. For biomechanical properties, evaluations include Young's Modulus, Shear Modulus, and flexural strength, utilizing techniques such as nanoindentation, dynamic mechanical analysis, and three-point bending tests.

## Data Availability

The data is available to the corresponding author upon request.

## Abbreviations

3-MPTS: 3-Methacryloxypropyltriethoxysilane
AEAPTMS: N-(2-Aminoethyl)-3-aminopropyltrimethoxysilane
AIRs: Ambiguous Interaction Restraints
Bis-GMA: Bisphenol A glycidyl methacrylate
EBPADMA: Ethoxylated bisphenol A dimethacrylate
FDA: Food and drug administration
HEMA: 2-Hydroxyethyl methacrylate
ICPTES: Isocyanatopropyltriethoxysilane
MD: Molecular dynamics
MTA: Mineral trioxide aggregate
NPT: Number of particles, system pressure, and temperature
NVT: Number of particles, system volume, and temperature
PME: Particle mesh Ewald
SDF: Structure data format
TEGDMA: Triethylene glycol dimethacrylate
TRIS: 3-(Trimethoxysilyl)propyl methacrylate
UDMA: Urethane dimethacrylate
VTES: Vinyltriethoxysilane

## References

[1] Ferracane JL (2011). Resin composite–state of the art. Dent Mater.

[2] Cho K (2022). Dental resin composites: A review on materials to product realizations. Compos B Eng.

[3] Mousavinasab SM (2011). Biocompatibility of composite resins. Dent Res J (Isfahan).

[4] Bertassoni LE (2011). Mechanical recovery of dentin following remineralization in vitro–an indentation study. J Biomech.

[5] Stansbury J (2005). Conversion-dependent shrinkage stress and strain in dental resins and composites. Dental materials : official publication of the Academy of Dental Materials.

[6] Perdigão J (2020). Current perspectives on dental adhesion: (1) Dentin adhesion - not there yet. Jpn Dent Sci Rev.

[7] Drummond JL (2008). Degradation, fatigue, and failure of resin dental composite materials. J Dent Res.

[8] Mitra SB, Wu D, Holmes BN (2003). An application of nanotechnology in advanced dental materials. J Am Dent Assoc.

[9] Jaberi Ansari Z, Kalantar Motamedi M (2014). Microleakage of two self-adhesive cements in the enamel and dentin after 24 hours and two months. J Dent (Tehran)..

[10] Mohanty PR (2023). Optimizing Adhesive Bonding to Caries Affected Dentin: A Comprehensive Systematic Review and Meta-Analysis of Dental Adhesive Strategies following Chemo-Mechanical Caries Removal. Appl Sci.

[11] Farooq I, et al. Synergistic Effect of Bioactive Inorganic Fillers in Enhancing Properties of Dentin Adhesives-A Review. Polymers (Basel). 2021;13(13):2169. 10.3390/polym13132169 PMC8271823 34209016

[12] Akhtar K (2021). Calcium hydroxyapatite nanoparticles as a reinforcement filler in dental resin nanocomposite. J Mater Sci Mater Med.

[13] Taira M (1993). Studies on radiopaque composites containing ZrO2-SiO2 fillers prepared by the sol-gel process. Dent Mater.

[14] Kaur K, et al. Comparison between Restorative Materials for Pulpotomised Deciduous Molars: A Randomized Clinical Study. Children (Basel). 2023;10(2):284. 10.3390/children10020284 PMC9955046 36832414

[15] Zhang YR (2014). Review of research on the mechanical properties of the human tooth. Int J Oral Sci.

[16] Galo R (2015). Hardness and modulus of elasticity of primary and permanent teeth after wear against different dental materials. Eur J Dent.

[17] Saini RS, Mosaddad SA, Heboyan A (2023). Application of density functional theory for evaluating the mechanical properties and structural stability of dental implant materials. BMC Oral Health.

[18] Alshadidi AAF (2023). Investigation on the Application of Artificial Intelligence in Prosthodontics. Appl Sci.

[19] Chiba A, et al. The influence of elastic moduli of core materials on shear stress distributions at the adhesive interface in resin built-up teeth. Dent Mater J. 2017;36(1):95–102. 10.4012/dmj.2016-160 28090032

[20] Rabelo Ribeiro JC (2008). Shear strength evaluation of composite-composite resin associations. J Dent.

[21] Zhu, H., et al. Effect of Shear Modulus on the Inflation Deformation of Parachutes Based on Fluid-Structure Interaction Simulation. Sustainability, 2023. 15. 10.3390/su15065396.

[22] Moradi Z (2020). Fracture Toughness Comparison of Three Indirect Composite Resins Using 4-Point Flexural Strength Method. Eur J Dent.

[23] Moosavi H, Zeynali M, Pour ZH (2012). Fracture Resistance of Premolars Restored by Various Types and Placement Techniques of Resin Composites. International Journal of Dentistry.

[24] Abdul-Monem M, El G, Al-Abbassy F (2016). Effect Of Aging On The Flexural Strength And Fracture Toughness Of A Fiber Reinforced Composite Resin Versus Two Nanohybrid Composite Resins. Alex Dent J.

[25] Durrant JD, McCammon JA (2011). Molecular dynamics simulations and drug discovery. BMC Biol.

[26] Kitchen DB (2004). Docking and scoring in virtual screening for drug discovery: methods and applications. Nat Rev Drug Discovery.

[27] Zhou X (2019). Development and status of resin composite as dental restorative materials. J Appl Polym Sci.

[28] Badar MS, Shamsi S, Ahmed J, Alam MA. Molecular Dynamics Simulations: Concept, Methods, and Applications. In: Rezaei N, editor. Transdisciplinarity. Cham: Springer International Publishing; 2022. p. 131–51.

[29] Elshereksi N, et al. Review of titanate coupling agents and their application for dental composite fabrication. Dent Mater J. 2017;36(5):539–552. 10.4012/dmj.2016-014 28652551

[30] Su H-L (2007). Silica nanoparticles modified with vinyltriethoxysilane and their copolymerization with N, N′-bismaleimide-4,4′-diphenylmethane. J Appl Polym Sci.

[31] Cousins KR (2011). Computer Review of ChemDraw Ultra 12.0. J Am Chem Soc.

[32] Dominguez C, Boelens R, Bonvin AMJJ (2003). HADDOCK: A Protein−Protein Docking Approach Based on Biochemical or Biophysical Information. J Am Chem Soc.

[33] Sun H (2016). COMPASS II: extended coverage for polymer and drug-like molecule databases. J Mol Model.

[34] Simmonett AC, Brooks BR (2021). A compression strategy for particle mesh Ewald theory. J Chem Phys.

[35] Cervino G (2020). Mineral Trioxide Aggregate Applications in Endodontics: A Review. Eur J Dent.

[36] Zhao H, Qi N, Li Y (2019). Interaction between polysaccharide monomer and SiO2/Al2O3/CaCO3 surfaces: A DFT theoretical study. Appl Surf Sci.

[37] Indumathy, B., et al. A Comprehensive Review on Processing, Development and Applications of Organofunctional Silanes and Silane-Based Hyperbranched Polymers. Polymers, 2023. 15. 10.3390/polym15112517. 10.3390/polym15112517 PMC10255420 37299316

[38] Jesionowski T, Krysztafkiewicz A (2001). Influence of Silane Coupling Agents on Surface Properties of Precipitated Silicas. Appl Surf Sci.

[39] Dermawan D, Prabowo BA, Rakhmadina CA (2021). In silico study of medicinal plants with cyclodextrin inclusion complex as the potential inhibitors against SARS-CoV-2 main protease (Mpro) and spike (S) receptor. Inform Med Unlocked.

[40] Brakat, A. and H. Zhu From Forces to Assemblies: van der Waals Forces-Driven Assemblies in Anisotropic Quasi-2D Graphene and Quasi-1D Nanocellulose Heterointerfaces towards Quasi-3D Nanoarchitecture. Nanomaterials, 2023. 13. 10.3390/nano13172399. 10.3390/nano13172399 PMC10489977 37686907

[41] Crouch J (2022). How van der Waals Approximation Methods Affect Activation Barriers of Cyclohexene Hydrogenation over a Pd Surface. ACS Engineering Au.

[42] Sahay S (2020). Role of Vander Waals and Electrostatic Energy in Binding of Drugs with GP120: Free Energy Calculation using MMGBSA Method. Journal of Shanghai Jiaotong University (Science).

[43] Erbaş A, de la Cruz MO, Marko JF (2018). Effects of electrostatic interactions on ligand dissociation kinetics. Phys Rev E.

[44] Li Z (2022). Electrostatic Contributions to the Binding Free Energy of Nicotine to the Acetylcholine Binding Protein. J Phys Chem B.

[45] Browning C (2007). Critical Role of Desolvation in the Binding of 20-Hydroxyecdysone to the Ecdysone Receptor*. J Biol Chem.

[46] Mondal J, Friesner R, Berne B (2014). Role of Desolvation in Thermodynamics and Kinetics of Ligand Binding to a Kinase. J Chem Theory Comput.

[47] Vaidya A, Pathak K. 17 - Mechanical stability of dental materials. In: Asiri AM, Inamuddin, Mohammad A, editors. Applications of Nanocomposite Materials in Dentistry: Woodhead Publishing; 2019. p. 285–305.

[48] Albergaria LS (2023). Effect of nanofibers as reinforcement on resin-based dental materials: A systematic review of in vitro studies. Japanese Dental Science Review.

[49] Fugolin AP (2020). Alternative monomer for BisGMA-free resin composites formulations. Dent Mater.

[50] Ribeiro JCR (2008). Shear strength evaluation of composite–composite resin associations. J Dent.

[51] Meenakumari C (2018). Evaluation of Mechanical Properties of Newer Nanoposterior Restorative Resin Composites: An In vitro Study. Contemp Clin Dent.

[52] Visan, A.I. and I. Negut Integrating Artificial Intelligence for Drug Discovery in the Context of Revolutionizing Drug Delivery. Life, 2024. 14. 10.3390/life14020233. 10.3390/life14020233 PMC10890405 38398742

[53] Meng XY (2011). Molecular docking: a powerful approach for structure-based drug discovery. Curr Comput Aided Drug Des.

[54] Jiang, B., et al. Molecular Dynamics Simulation on the Interfacial Behavior of Over-Molded Hybrid Fiber Reinforced Thermoplastic Composites. Polymers, 2020. 12. 10.3390/polym12061270. 10.3390/polym12061270 PMC7361982 32498238

[55] Barbhuiya S, Das BB (2023). Molecular dynamics simulation in concrete research: A systematic review of techniques, models and future directions. Journal of Building Engineering.

[56] Gähde U, Hartmann S, Wolf J. Models, Simulations, and the Reduction of Complexity. Berlin, Boston: De Gruyter; 2014. 10.1515/9783110313680.

[57] Marshall GR (2013). Limiting assumptions in molecular modeling: electrostatics. J Comput Aided Mol Des.

[58] Schmitt S, Fleckenstein F, Hasse H, Stephan S. Comparison of Force Fields for the Prediction of Thermophysical Properties of Long Linear and Branched Alkanes. J Phys Chem B. 2023;127(8):1789–1802. 10.1021/acs.jpcb.2c07997 36802607

[59] Zhou W, et al. Modifying Adhesive Materials to Improve the Longevity of Resinous Restorations. Int J Mol Sci. 2019;20(3):723. 10.3390/ijms20030723 PMC6387348 30744026

[60] Carvalho RM (2012). Durability of bonds and clinical success of adhesive restorations. Dent Mater.

[61] Ouldyerou A (2023). Biomechanical performance of resin composite on dental tissue restoration: A finite element analysis. PLoS ONE.

[62] Elleuch S (2023). Agglomeration effect on biomechanical performance of CNT-reinforced dental implant using micromechanics-based approach. J Mech Behav Biomed Mater.

[63] Chopra D (2024). Load, unload and repeat: Understanding the mechanical characteristics of zirconia in dentistry. Dent Mater.

[64] Sachan S (2024). In Vitro Analysis of Outcome Differences Between Repairing and Replacing Broken Dental Restorations. Cureus.

